# Phenotypic Novelty in EvoDevo: The Distinction Between Continuous and Discontinuous Variation and Its Importance in Evolutionary Theory

**DOI:** 10.1007/s11692-016-9372-9

**Published:** 2016-04-28

**Authors:** Tim Peterson, Gerd B. Müller

**Affiliations:** 1 Department of Theoretical Biology, University of Vienna, Althanstrasse 14, 1090 Vienna, Austria; 2 The KLI Institute, Martinstrasse 12, 3400 Klosterneuburg, Austria

**Keywords:** Evolutionary theory, EvoDevo, Phenotypic novelty, Innovation, Macroevolution

## Abstract

The introduction of novel phenotypic structures is one of the most significant aspects of organismal evolution. Yet the concept of evolutionary novelty is used with drastically different connotations in various fields of research, and debate exists about whether novelties represent features that are distinct from standard forms of phenotypic variation. This article contrasts four separate uses for novelty in genetics, population genetics, morphology, and behavioral science, before establishing how novelties are used in evolutionary developmental biology (EvoDevo). In particular, it is detailed how an EvoDevo-specific research approach to novelty produces insight distinct from other fields, gives the concept explanatory power with predictive capacities, and brings new consequences to evolutionary theory. This includes the outlining of research strategies that draw attention to productive areas of inquiry, such as threshold dynamics in development. It is argued that an EvoDevo-based approach to novelty is inherently mechanistic, treats the phenotype as an agent with generative potential, and prompts a distinction between continuous and discontinuous variation in evolutionary theory.

## Introduction

Different concepts reflect different priorities in research programs (Wagner 2014)

The biological concept of “novelty” has various applications depending upon which field is utilizing the term. As Wagner points out, there is nothing inherently wrong with this. The view of what a novelty is varies according to the requirements of each field in order to make the term functional. However, while novelties have long been considered an important and neglected problem in evolutionary theory (Mayr 1960), there is debate on whether they are distinct from continuous, adaptational change (Love 2003; Müller and Newman 2005). Although the existence of structures that are not present in ancestral groups is a biological reality, how these structures originate and how they are accounted for in evolutionary theory is a topic of discussion.

At the center of the issue is the question of whether morphological evolution proceeds purely by the accumulation of quantitative variation, with any changes that are qualitative appear as a consequence of the accumulation of small alterations; or whether there are instances of discontinuous change that are mechanistically different from continuous modifications, and cannot be extrapolated from the summation of adaptations. The mechanisms underlying discontinuous changes may also affect the likelihood of trait retention and its spread in a population (West-Eberhard 2003). This relates to a corollary problem on the consequences of morphological novelty origination in phenotypic evolution. If these novelties are a subset of continuous change, their appearance is likely explained by selection on a new function combined with, perhaps, innovation at the genetic level. However, if morphological novelties represent discontinuous events of change resulting from higher level processes, selection cannot be invoked without resorting to circular arguments (Moczek 2008). Instead, novelties would represent unrefined variational additions for selection to act on.

This notion of discontinuity is common in usage of the term across various fields of research and indicates a conceptual distinction from standard variation. Some commentators have played down this importance, arguing that novelty is essentially another term for variation or a subset of variational change (Arthur 2000), whereas other accounts emphasize that novelties represent a distinct class of evolutionary change (Müller and Wagner 1991; Wagner 2014). This article details how novelties are studied in evolutionary developmental biology (EvoDevo), particularly at the level of the phenotype, and how they represent autonomous biological entities. Potential practical applications of the novelty concept and implications that have been sidelined in evolutionary theory are equally addressed. This is crucial for giving significance to the concept, as it is too often weighed down in arguments over definitions. To contrast novelty in EvoDevo with uses from other fields, an introductory description of how the novelty concept is employed by geneticists, population geneticists, morphologists, and behavioral biologists is provided. Though each field has its own terminology, and new traits are not always explicitly stated using the word “novelties,” each of these fields offers a means for dealing with traits that were not present in ancestral species. Although the present study relies predominantly on animal examples, plants show an equally broad distribution of novelties across all taxa. The general implications of the phenotypic novelty concept apply to plants as well.

Often the idea of novelty is treated in papers describing what “novelty” is, or how it is outside the scope of population genetics (Müller and Newman 2005; Pigliucci 2008; Hallgrímsson et al. 2012). While these advances are helpful in their own right, here the concept is taken beyond the descriptive realm or a definitional debate. Practical guidelines and detailed examples are given to show how an EvoDevo-specific approach to novelty can be used in experiments, modeling, databank creation, and more. It is also addressed how this strategy is productive for the advancement of evolutionary theory. Specifically, three themes about researching novel phenotypes in an EvoDevo context are discussed: (1) The generative potential, explanatory power, and predictability of different kinds of novelty generation, (2) The distinction of discontinuous and continuous change of structural traits, (3) The role of novelty generation in evolutionary theory.

These themes indicate how the novelty concept can be used for research in more than a descriptive manner. Crucially, the EvoDevo approach to phenotypic novelty seeks to provide a mechanistic explanation of morphological change. This reinforces recent suggestions that EvoDevo has explanatory power, despite this potential often being attributed solely to (population) genetics (Gilbert et al. 1996; Wagner 2000). These insights are not meant to replace, or modify, the ideas or practices found in other fields. Instead, they relate to events that fall outside of the priorities of other research programs.

## Novelty Usages Outside of EvoDevo

While novelty and innovation are major concerns also in fields outside of biology, such as cultural (O’Brien and Shennan 2010; Charbonneau 2015), technical (Krohs and Kroes 2009), economic (Nelson 2009), or linguistic (Számadó and Szathmáry 2006; Steels 2011) systems, for the purpose of the present paper we differentiate several usages of novelty in biology.

### Genetics

Use of “novelty” in genetic studies is often related to the debate about what a “gene” is. Recent discoveries concerning gene location and structure, epigenetics, chromosome structure, post-transcriptional and post-translational events, structural variations, along with pseudogenes and retrogenes, have made the concept far less clear than it was during the formulation of the Modern Synthesis (Gerstein et al. 2007). Various authors have proposed definitions for the gene (Pearson 2006; Gerstein et al. 2007; Pesole 2008). Though none are considered as a standard, the most cited definition is from Gerstein et al.: “a union of genomic sequences encoding a coherent set of potentially overlapping functional products.” Current definitions, such as the above, have replaced the original view of genes as discrete inherited effects with discrete physical characters. In the new context, “novel gene” is used most often for communication of the content of a paper, namely that the authors are describing a gene as a physical entity that has been newly identified or when a new function was discovered for a previously known gene (Steeg et al. 1988; MacDonald et al. 1993; Fleury et al. 1997; Nishimura et al. 2014; Agaram et al. 2015; Mangino et al. 2015). Articles that identify a specific “novel gene” typically do not focus on the mechanisms behind the emergence of genes or comment on theoretical concerns about their origins. Instead, they concentrate on the function or phylogenetic context of the gene being discussed. This may include information on cis-regulatory elements, which function as genes in the sense of inherited effects on the phenotype but are not included in all definitions of genes.

In contrast, a number of articles approach the concept of a novel gene origination by examining processes such as duplication, combinations, and de novo generation. Duplication has long been considered a source for the origination of new genes that occurs regularly (Ohno 1970; Kimura 1983). The resultant genetic redundancy permits the accumulation of mutations in the new gene, leading to its loss (the “most likely fate”), to the acquisition of a new function, or to sub-functionalization (Prince and Pickett 2002; Conant and Wolfe 2008). While it has been argued that divergence without loss only happens in genes that began with bifunctionality (Hughes 1994), it is possible for genes to be expressed in new locations after changes in their cis-regulatory elements (Rebeiz et al. 2011). Gu has developed a model for estimating the degree to which duplicates have diverged (Gu 1999), while Walsh created a formula for determining the likelihood of the gene gaining a new function based on population size and mutational advantage (Walsh 1995). On the other hand, divergence may be prevented if there is selection for higher quantities of the gene product (Thomas 1993) or if there is feedback between the duplicates (Kafri et al. 2009). The importance of selection for duplications and the likelihood of a new function can be modeled (Ohta 1986; Lynch and Katju 2004), and the outcomes of novel duplications have been assessed (Zhang 2003; Francino 2005). While retained duplicates are novel units at the genetic level, as they only impact copy-number variation, the potential that both genes will be retained while diverging does not necessarily create an increased probability for discontinuous phenotypic change compared to existing genes. This is one example of the semi-independence of genetic and phenotypic novelties.

Another mechanism for the origination of novel genes is the combination of two or more transcriptional regions. This can occur due to the insertion of transposable elements (McClintock 1948; Nekrutenko and Li 2001; Lorenc and Makałowski 2003), through gene rearrangements (Early et al. 1980), or via the introduction of a gene by horizontal gene transfer (Bergthorsson et al. 2003). It may also occur during transcription if two adjacent genes are transcribed together in what is known as tandem chimerism (Parra et al. 2006). Post-translational events may also cause two previous genes to be considered a novel gene, since unrelated mRNA (Borst 1986) or proteins (Handa et al. 1996) may be spliced together. Since recent definitions of the gene include the ability for multiple gene products (Pearson 2006; Gerstein et al. 2007; Pesole 2008), it is possible that novel gene origination from new combinations would not result in the elimination of previous gene functions. It has been suggested that the origination of new genes from combinatorial events may impact the fluctuating rates of evolution (Zeh et al. 2009) and explain punctuated equilibrium (Gould and Eldredge 1977).

Novel genes have also been shown to arise de novo from non-coding regions. This has been seen in *D. melanogaster*, particularly when linked to selection pressure (Levine et al. 2006). The conversion of non-coding regions to exons is sometimes associated with mutations in pre-existing genes and amplifications of short sequences, as found in antifreeze proteins of some arctic fish (Chen et al. 1997a, b). The rate at which de novo origination may occur can be high. In a study on various *Drosophila* species, 11.9 % of the new genes found were created de novo (Zhou et al. 2008). Many of the ways by which new genes can form, and the impact from these genes on the resultant phenotype, are described by Kaessmann (2010).

### Population Genetics

In the field of population genetics, novelty oriented research examines how new genes or alleles spread in a population and what consequences their introduction has in evolutionary terms. “New gene” and “novel gene” are mostly used synonymously. Andreas Wagner has written an influential book on innovation in population genetics (Wagner 2011). While avoiding an in-depth definition of novelty, it is argued that novelties are a “new feature that endows its bearer with qualitatively new, often game-changing abilities.” These are divided into three classes: Metabolic networks, regulatory circuits, and proteins or RNA molecules. More macroscopic novelties are stated to be dependent on these. The focus is placed on the gene pools of populations and on how numerous genotypes can result in the same phenotype, creating “genotype networks.” Neutral mutations possible within these genotype networks grant robustness of the phenotype. This, it is argued, permits the exploration of genotype space in order to find configurations that create novelties.

The understanding of novelty within population genetics is still impacted by the various definitions for genes. However, as the focus is on processes, such as the searching of genotype space by genotype networks, many of the issues in defining genes are alleviated. Focus on the process also permits population geneticists to work with both real world cases as well as mathematical models in which selection, mutation, and fitness can be controlled. As a consequence, population genetics is concerned with the theoretical issues involved in the introduction of a new gene, for example relative timing of changing selection pressures versus novel mutations (Wright 1932) or stable states of genetic redundancy from new genes with the same function (Nowak et al. 1997).

These theoretical problems are more easily studied by modeling populations. One important question about novel genes examined at the population level is whether adaption to a new environment comes from selection on pre-existing genetic variation or from novel genes (Barrett and Schluter 2008). Another issue for population genetics is the role of demes in generating novel genes or chromosomal configurations that can spread through a population (Wright 1931). This includes modeling, for example detailing the spread of novel mutations through demes (Lande 1979), as well as critique of the role of local populations in producing complex novelties (Coyne et al. 1997). A further usage that has become commonplace in population genetics is the likelihood of fixation or loss of a new gene, based on factors such as epistasis, selection pressure, and population size, among others.

An issue that population genetics faces when determining selection on novel genes, or how these novel genes may impact the phenotype, is that new genomic sequences may be modifiers of existing genes (Merlo and Boyle 2003), and may function via the release of cryptic variation (Gibson and Dworkin 2004). It appears to be a general rule that the genetic background can intensify or conceal new alleles (Polaczyk et al. 1998; Gibson et al. 1999), which may confound accurate assessment of the role of novel genes.

### Morphology

The treatment of novelty in morphology is tightly interconnected with the concept of homology, which has experienced significant debate (Tautz 1998; Hall 1999; Wagner 2014). While genes propagate using the ancestor as a template, there is a lack of direct continuity between generations of morphological structures, which are built anew in each individual. Further complicating the issue, quantification of such structures is fraught with problems, as the traits differ between individuals and change over a lifespan. Trying to circumvent these problems by identifying homologous traits through their development or the genes coding for them is hampered by developmental systems drift (True and Haag 2001) and autonomization (Müller 2003). One possible solution that has been suggested is that persistent regulatory networks may code for character identity, while other genes regulate character state (Wagner 2014) through genotype networks/neutral genotypes (van Nimwegen et al. 1999; Dall’Olio et al. 2014).

Still, the terminology of novelty is often used without major problems, particularly in paleontology. There are three typical uses of novelty in morphology that overlap to some degree, and often more than one of these themes is included within a single paper. One of the more common ways in which novelties are evoked is to convey that a discrete structural unit (homologue) or body plan appears in a phylogenetic lineage, and in describing the new trait which can be helpful in distinguishing different species (Schopf and Morris 1994; Schweitzer et al. 2004; O’Keefe et al. 2011; Holliday and Gardner 2012). The concept is also used to generate questions about adaptational events in evolution, such as what selection regimes may have led to the novelty, what functional uses it permitted, or what its role in speciation may have been (Erwin 2000; Carroll 2001; Hou et al. 2004; Nielsen and Parker 2010). Though too strict of an adaptationist stance has fallen out of style as of late, this usage is often helpful in identifying structures that are “key innovations” (Liem 1973; Love 2003). Lastly, novelties are used to infer generalizations about evolution, for example where and when the introduction of novel morphological characters is most likely to occur (Jablonski et al. 2006; Marshall 2006; Jablonski 2007; Budd and Pandolfi 2010), whether this is impacted by the presence of empty ecological niches (Valentine 1981; Odling-Smee et al. 2003; Valentine 2004) or how novelties themselves change the ecosystems carrying capacity (Erwin 2012), and if there is lag present between novelty origination and adaptive radiation (Erwin 2015). A key time period examined is the Cambrian explosion with its rich array of novel body plans.

Paleontological studies are frequently combined with information from other fields (Sepkoski and Ruse 2009), and this is particularly true in the case of novelties. Molecular or developmental research on extant species supplements the fossil evidence in determining what changes led to the formation of a novel structure (Shubin et al. 1997; Shubin 2002; Ruta et al. 2006). While this kind of association may be hampered to some degree by developmental systems drift (True and Haag 2001), it still provides a powerful combination for understanding the development of extinct species and how morphological novelties arose in various lineages.

### Behavior

Behavioral studies often attempt to understand the origin and integration of a new behavior in a population and the resulting consequences (Reader and Laland 2003). This can be difficult to assess, due to the infrequency of appearances and problems with quantifying learning, cognition, and social behavior. As such, most studies on new behaviors focus on responses to man made events or captivity (Ramsey et al. 2007). Therefore, the most common use is the documentation of new behaviors, e.g., novel feeding behaviors in birds (Lefebvre et al. 1997). Instances of new types of behavior are recorded together with the environment in which the behavior occurred and with physical attributes of the organisms. These factors are taken to be helpful in determining the causes of new behaviors.

Behavioral innovation may depend on morphological changes but may also itself induce morphological change (West-Eberhard 2005). In the case of a two-legged goat born without forelegs, the novel behavior of moving on two legs results in a series of changes to the skeletal structure and the associated musculature (Jiang et al. 2003). Similarly, behavioral innovation can prime a species for morphological innovation (Mayr 1960). The first instance of a structure might not have an adaptive advantage since it cannot, by definition, have been selected for. However, if a new behavior arises, then any morphological novelty that appears later can be “used” by this behavior and may be retained. This can be seen in sexual selection for bristle contact during copulation that is present in sepsid flies. Some male sepsid flies have bristles on the fourth sternite, and a subset of these species have moveable appendages that allow some control of the bristles (Eberhard 2001). In cases where the preference for bristle contact during copulation was present, morphological novelties such as moveable sternites that increase the contact were selected for and retained. This preference was necessary for the introduction of the novel phenotype but not sufficient (Wagner and Müller 2002). In this way novel behaviors may guide which random mutations will be positively selected for (Wcislo 1989).

## Novelty in EvoDevo

Phenotypic novelty has been called a core tenet of EvoDevo (Love 2006), and several articles have been addressing how novelties should be classified or used in EvoDevo (Müller 1990; Müller and Wagner 1991; Pigliucci 2008; Hallgrímsson et al. 2012; Peterson and Müller 2013; Wagner 2014). Development interconnects many evolutionarily relevant factors, for example genetic regulation, higher order tissue interactions, patterning mechanisms, physical forces, and environmental influences. As a result, EvoDevo is a broad field that permits the inclusion of several uses for the concept of novelty. Wagner (Wagner 2014), for instance, highlights the view of novelties based on gene regulatory networks and how they can guide future research. Others emphasize developmental dynamics or epigenetic factors (Newman and Müller 2000; West-Eberhard 2003; Maresca and Schwartz 2006).

Here we examine experimental and modeling applications that are used in view of novelty research that is preferentially couched in the levels of cell and tissue interactions, or organ and organ system formation. This has the benefit of adhering more closely to how the term is commonly understood in reference to structures, such as new body plans, bones, shells, muscles, horns, or other phenotypic units (Mayr 1960; Liem 1973; Müller 1990; Arthur 2000; West-Eberhard 2003; Cebra-Thomas et al. 2005; Hall 2005a; Newman and Müller 2005; Moczek et al. 2007; Pigliucci 2008; Müller 2010; Leys and Riesgo 2012). In addition, it can address events typically overlooked by more gene-oriented programs. This requires an elaboration of previous work (Müller 2010; Peterson and Müller 2013), in which a type 1 novelty (T1) refers to a new primary body assemblage, a type 2 novelty (T2) refers to a structural element with no homologous counterpart in the ancestral species, and a type 3 novelty (T3) refers to a unit or character that has been incrementally individualized due to variation in a new direction or dimension that was not previously possible (see Table 1 for examples). This can be summarized by the following improved definition:

Phenotypic novelty refers to a primary body plan (T1), new constructional element (T2), or newly individualized character (T3) that is qualitatively discontinuous from the ancestral state.

**Table 1:** Examples of phenotypic novelties

Novelty	Novelty type	Justification	References
Cell	T1	Original body plan	Trevors (2003)
Metazoan bodyplans	T1	Original multicellular body plans	Newman and Bhat (2008)
Extra digits in Maine Coon felines	T2	Discrete new homonomous element	Lange et al. (2014)
Joint in cichlid pharyngeal jaws	T2	New cartilaginous element between the skull and jaws	Liem (1973) and Galis and Drucker (1996)
Tissues for carapace and plastron in turtles	T2	Introduction of dermal bones	Burke (1989), Gilbert et al. (2001) and Nagashima et al. (2007)
Horns of dung beetles	T2	Switch from transient juvenile structure to adult trait	Moczek (2005)
Teeth in vertebrates	T2	Introduction of a tissue type	Smith and Coates (1998)
Lantern of fireflies	T2	Organ with new photic layer	Stansbury and Moczek (2014)
Feathers in avians	T2	Switch from planar scales to tubular feathers	Prum and Brush (2002)
Joint in Anuran jaw	T2	New cartilaginous element in tadpoles	Svensson and Haas (2005)
Internal cheek pouch of Geomyoid rodents	T2	Switch from external pouch to a fur lined internal pouch	Brylski and Hall (1988a, b)
Joint in bolyrine snakes jaws	T2	New cartilaginous element in the maxilla	Frazzetta (1975, 2011)
Fibular crest in Theropods	T2	Insertion of a bone sesamoid which fuses to a preexisting structure	Müller and Streicher (1989)
Wing scales in Lepidoptera	T2	Switch from sensory bristles to cuticular scales on the wings	Beldade and Brakefield (2002)
Moveable abdominal appendages in sepsid flies	T2	Novel appendages from histoblasts	Bowsher and Nijhout (2007)
Carpel of flowering plants	T2	Fusion of cupule tissue forming the carpel	Cronk et al. (2002)
Wing-hearts in insects	T2	Switch of pericardial cell lineage into a wing circulatory organ	Pass et al. (2015)
Tusk in Narwhals	T3	Rostral and rotational growth of canine	Nweeia et al. (2012)
Pronotum of treehoppers	T3	Growth of the pronotum in new dimensions	Stegmann (1998) and Yoshizawa (2012)
False thumb in pandas	T3	Elongation of a bone sesamoid into a false finger	Abella et al. (2015)
Corpus callosum in humans	T3	Links the two forebrain hemispheres without traveling through the lamina terminalis	Mihrshahi (2006) and Wagner (2014)
Odontoid processes in dracula fish	T3	Elongated bones forming false teeth	Britz et al. (2009)
Nasal appendages of the star-nose mole	T3	Epidermal outgrowths of the nose	Catania et al. (1999)
Yolk sec extension in Cypriniform fishes	T3	Ventral, linear elongation of the yolk sac	Virta and Cooper (2009)
Prickles of plants	T3	Sharp extensions of the epidermis	Niklas (1997)

Examples of different categories of phenotypic novelties based on the definition used in this article, referring to *a primary body plan (T1), a new constructional element (T2), or a newly individualized character (T3) that is qualitatively discontinuous from the ancestral state*. Justifications for each novelty are listed along with a supporting reference

The implied difference between continuous and discontinuous variation requires elaboration. Continuous variation of a trait refers to changes in a quantifiable property across extensive numbers of generations. Discontinuous variation refers to a binary change between the two states of absent and present. Innovation (in this usage) is the process by which discontinuous variation switches to the new state, with novelty referring to the resultant structure. A simplified example may help with clarity: A bone that exists in both the F1 and F2 generations has continuous variation due to changes in properties such as length, diameter of the shaft, Young’s modulus, etc. Discontinuous variation is a bone that did not exist in the F1 generation, but is present in the F2 generation. The above definition delineates three kinds of discontinuous change based on their innovation and the resultant novelty, with each described in detail below. While T1 and T3 novelties are included, the main focus of the present article is on T2 novelties.

A principal caveat seems indicated here. Though the definition provided above may be applicable at biological levels of organization below tissues (i.e., cells, proteins, genes, etc.), novelty at one level (e.g., a new gene) should not be used to determine novelty at another level (e.g., a new morphological structure). This is due to the loose causal connections between levels of organization. Over time the adult phenotype of a tissue expressed in a lineage can become decoupled from its original development or underlying genes (Hall 1984; Wray and Raff 1991; Patel 1994; Shubin and Alberch 1994; Sommer and Sternberg 1996; Wray and Abouheif 1998; Félix 1999; Wray 1999; Wray and Raff 1999; Félix et al. 2000; Butler and Saidel 2000; Salazar-Ciudad and Jernvall 2002; Müller 2003), and developmental systems drift appears to be a ubiquitous phenomenon (Hall 1984; Wray and Raff 1991; Patel 1994; Sommer and Sternberg 1996; Félix 1999; Wray and Raff 1999; True and Haag 2001; Salazar-Ciudad and Jernvall 2002). This can lead to phenotypic structures that vary only quantitatively with obvious historical continuity, which nonetheless have differing developmental or genetic underpinnings in the extant and ancestral conditions. Examples include the autopod of urodele salamanders, which develop in the opposite sequence from other tetrapods (Gardiner et al. 1998), and the conserved vulva of nematodes, which has multiple patterns of development (Sommer and Sternberg 1996; Félix 1999; Félix et al. 2000). This results in homologous phenotypes that differ in how they are derived. Therefore, homologues can be thought of as organizers of the phenotype that have a level of autonomy from their genetic and developmental underpinnings (Müller 2003). Because of this, it is important to distinguish what type of novelty is being described. This can be accomplished with a simple modifier, such as “novel gene” or “novel tissue.” The authors have made this suggestion before (Peterson and Müller 2013), and the need for clear distinction has been asserted by others (Moczek 2008).

### Type 1: Primary Body Assemblages

The most foundational level of a discontinuous phenotypic change is the establishment of new body assemblies—often called “body plans” in their permanent forms. This sets up the infrastructure upon which other novelties can be added to and modified over time. In previous work on novelties, this class of novelty had been ascribed to multicellular assemblages alone (Müller 2010; Peterson and Müller 2013). However, the initial introduction of the cell itself should also be considered a T1 novelty, as it set a foundational body plan for unicellular organisms that could evolve and add other units such as the cytoskeleton, flagella, or ribosomes. The same applies to the origin of the egg cell (Newman 2011). Key steps in the evolution of life termed “major transitions” (Maynard Smith and Szathmáry 1998) also conform with this view of novelty.

The study of Type 1 novelties can be divided into three major issues: the origin of life (abiogenesis), multicellularity origination, and multicellular body plan origination. The first of these, the development of cellular life, is an unsolved question, and there is an entire field dedicated to understanding how it may have occurred. Dozens of books have been written on the subject without a clear answer, however it can be said that the chemico-physical properties of the molecules involved are a central theme (Chakrabarti and Deamer 1992; Volkov et al. 1996; Luisi et al. 1999; Segré et al. 2001; Trevors 2003; Mansy et al. 2008; Zhu and Szostak 2009). The approach, therefore, will rely on understanding the chemical physics of the molecules involved in the production of (proto)cell components, such as cell membranes and RNA, and on an understanding of the environment and raw materials present when life first originated. Terry Deacon, for instance, has developed a model where autocatalytic activity produces self-assembling components that encapsulate the processes in structures of various shapes of tubes or polyhedrons (Deacon 2006).

Ideally, each component of organismal life (compartment formation, metabolism, etc.) can be approached separately to see under what conditions it could arise, providing us with clues as to the order of events that occurred and the circumstances that led to the appearance of each part. For example, it is known that lipid membranes can spontaneously form under certain conditions (Segré et al. 2001), and that this formation can be accelerated by certain minerals that may also catalyze RNA polymerization (Hanczyc et al. 2003). Efforts have been made to synthesize such protocells, which can inform about what requirements were needed to create life (Szostak et al. 2001; Blain and Szostak 2014). However, many questions still remain: does the formation of an RNA or DNA molecule require isolation from the surrounding environment? How do these molecules first form? How did they become self-replicating? These are questions that will require the further study of chemical physics and biochemistry.

It has been proposed that multicellular life began in a “pre-Mendelian world” in which the genotype-phenotype connection was much looser than it is today (Newman and Müller 2000; Newman 2005); the configuration of first multicellular assemblies would have been dominated by the physical properties of the cells involved, and simple patterning mechanisms, for example cellular adhesiveness, polarity, chemical oscillation, overall shape and size would have dictated the phenotypic outcomes (Niklas 2000; Newman et al. 2006; Newman and Bhat 2008). However, while unicellular organisms were able to aggregate due to their surface properties and facilitating environmental conditions (such as changes in Ca^2+^ levels) they would not be able to form complex shapes until the co-option of cadherins that previously had functions for single-celled organisms (Newman 2016). Assuming there was no tight connection between genotype and phenotype in these multicellular assemblages, selection acting on the phenotype would not “reach” the genotype to select for the retention of a particular set of genes. As such, an understanding of form and novelty in these early stages is only partially served by working with a gene-selection or population-centered perspective. The self-assembly of cells based on their phenotypic, biochemical, and physical properties provides a more informed understanding of the origin of simple body plans. Focusing on the traits of unicellular organisms, such as adhesiveness or cell signaling, can identify new modes of transformation to multicellularity (Niklas 2014).

With the establishment over time of a tighter genotype-phenotype relation, changes in the genome would have had a more consistent impact on the structural outcomes. At this point the developmental-genetic toolkit must have begun to play a greater role in body plan origination. New genetic changes, particularly in regulatory elements, can have caused shifts large enough to create new body plans. However, development consists of dynamical interactions that involve not only gene expression but also interactions between and among cells, tissues, and the physiochemical environment (Heegaard et al. 1999; Elder et al. 2000; Newman and Müller 2000; van der Meulen and Huiskes 2002; West-Eberhard 2003; Keller et al. 2003; Altenberg 2005; Gilbert 2005; Haudenschild et al. 2009; Grad et al. 2011). Therefore, the origin of T1 novelties requires a multidisciplinary understanding: how the developmental-genetic toolkit changes through duplications, deletions, and mutations; how changes early in ontogeny can be amplified as the organism develops; feedback loops between tissues and the genome; tissue to tissue interactions. Far from representing a complete list, this demonstrates that T1 novelties require a more thorough understanding than shifting allele frequencies or the genotype’s immediate products. Other authors have commented on the need for such an inclusive approach to the origin of multicellular life (Arnellos et al. 2013).

### Type 3: Individualized Unit Based on a New Dimension of Variation

The concept of “morphospace” was introduced in the mid 60’s along with the idea that only a portion of possible shapes within such a space is utilized (Raup 1966). The range of morphologies realized is constrained both by selection (Drake and Klingenberg 2010) and by development, the latter both historically (Gould and Lewontin 1979) and morphogenetically (Oster et al. 1988). Therefore continuous modification of traits is only possible along a finite number of axes, and any variation along a previously impossible axis represents a qualitative change that may trigger phenotypic novelty, despite the resultant structures having a homologous counterpart in the ancestral species. Examples of this may include the narwhal tusk, the moveable appendages of male sepsid flies, or the pronotum of many members of the insect family Membracidae.

The narwhal tusk is an extremely enlarged canine that has a new spiral pattern, causing it to protrude straight rostrally (Nweeia et al. 2012). This very straight outgrowth, which aids in hydrodynamics, would be impossible to form without the novel spiraling growth pattern (i.e., a new axis of variation), since any minor deviation would be amplified (Kingsley and Ramsay 1988). In sepsid flies, new variation in density of the medial area of a cuticular sclerite, combined with new associated musculature, has resulted in moveable nongenitalic appendages (Bowsher and Nijhout 2007). This structure was then further individualized for use in stimulating the female reproductive organs. In the Membracidae family, new variation in the pronotum has allowed this sclerite to be individualized into a wide range of diverse shapes (Yoshizawa 2012). These structures, which are often used for defense or camouflage, are not found in other groups (Stegmann 1998) and clearly represent structures (Moczek 2008) that fit the definition of T3 novelties.

T3 novelties can often be identified as elements that have been greatly individualized, particularly if only one structure of a serially homologous set has been drastically altered. However, it can be difficult to ensure that the new variation was not possible in the ancestral state. Caution is recommended when trying to determine the causes of individualization. Some cases may be easily discerned, such as when the underlying genetic architecture has been modified to permit a new direction of growth, but in other cases it may be less clear if the individualization represents a qualitatively new dimension.

A note is added here before concluding the discussion on T3 novelties. While discontinuity in T1 and T2 novelties refers to rapid change in the presence of the entire structure in a population and a binary switch in a single lineage, T3 discontinuity refers to the presence of variation in a new direction or dimension. Defining T3 novelties as has been done here maintains the principle of discontinuity, while allowing for structures that have homologous counterparts in the ancestral species to be included. This type of variation has been recommended before as part of the novelty concept (Hallgrímsson et al. 2012). The current authors previous stated view was that the “onset of adaptive advantage” could be used for specifying the origin of a T3 novelty.

This being said, the reliance on adaptive advantage for T3 novelties (Peterson and Müller 2013) creates several problems. Not all evolution is adaptive (Gould and Lewontin 1979; Alberch and Gale 1985; Lynch 2007; Koonin 2009), and the first appearance of a phenotypic element is even more unlikely to be adaptive, since it cannot have been selected for, or refined by, natural selection before its first appearance (Moczek 2008; Pigliucci and Müller 2010; Peterson and Müller 2013). This is not to say that novelties cannot be or cannot become functional, but those definitions based on functionality risk missing a majority of novelties. Furthermore, defining novelty through function restricts the concept to interactions of existing traits with the external environments rather than focusing on the traits themselves (Erwin 2015).

This problem is ameliorated by incorporating part of the definition by Hallgrímsson et al. (2012). Their novelty concept contained two criteria: a transition between adaptive peaks on a fitness landscape and breaking developmental constraints to generate variation in a new direction or dimension. While transitioning between adaptive peaks is possible without requiring that novelties arise with adaptive value, and is useful for the applications of Hallgrímsson’s approach, the definition in the current paper focuses on morphological traits and uses the second aspect, that morphological variation of the phenotypic structure in a new dimension is needed to qualify as a qualitative change. For T3 novelties, breaking of developmental constraints is likely the most common precondition that permits novel variation, though the two events are not equivalent. For example, genetic integration can cause two traits to be developmentally constrained from diverging (Wagner and Altenberg 1996). If this constraint is removed while selection continues to force the traits to co-vary, there will be no change in phenotypic structure despite the overcoming of the constraint. Furthermore, developmental constraints often depend on external factors, and therefore can be counteracted by changes in the environment (Sansom 2009), potentially leading to new variation without genetic change.

### Type 2: Element with No Homologous Counterpart in the Ancestral Species

T2 novelties focus on the introduction of new elements into an existing body plan. This characterization has been used before as a way to differentiate novelties from standard variational change (Müller and Wagner 1991; Pigliucci and Müller 2010; Peterson and Müller 2013) since it refers to a structure with no homologous counterpart in the ancestral species and therefore, by definition, cannot be a variation of another trait. Though at times considered too restrictive when used as the only type of novelty (Arthur 2000; Pigliucci 2008; Moczek 2008), this criticism has been diminished with the distinction of T1, T2, and T3 novelties. The importance of T2 novelties is centered on the capacity to introduce a new element instead of a quantitative alteration of previous structures. Significantly, T2 novelties show how the developmental system can play a generative role in evolution at the phenotypic level of biological organization. This is possible because development relies on local cues and subroutines that determine which genetic pathways are used at a position or time, and relies on interactions among cells, tissues, and the physiochemical environment (Heegaard et al. 1999; Elder et al. 2000; Newman and Müller 2000; van der Meulen and Huiskes 2002; West-Eberhard 2003; Keller et al. 2003; Altenberg 2005; Gilbert 2005; Haudenschild et al. 2009; Grad et al. 2011). This means that existing genetic networks can be co-opted to activate in new sites based on epigenetic signals such as tissue interactions or morphogen gradients. The use of the term “epigenetic” to describe these events occurring above the gene level is in line with how it was used before it became synonymous in some fields with non-DNA altering changes of the genome, for example DNA-methylation—representing a subset of the broader definition. The modular ability of such subroutines, with the potential to be summoned, permits the expression of coherent structures in new locations.

Type 2 novelties were suggested to be triggered by threshold effects of gene expression or of developmental interactions, the propagation of altered early stage conditions, the combination of preexisting structural units, or the retention of transient structures from ontogeny. Previous use of this concept restricted it to phenotypic character traits such as a new element of bone or shell. However, the principle of a discontinuous addition of a new element to an existing body plan is applicable to multiple organizational levels. In contrast to earlier understanding (Müller and Wagner 1991), here new serially homonomous structures are also considered novelties, as they can represent discontinuously new elements in the body plan.

While discontinuous additions are not restricted to phenotypic character traits, the co-option of genetic networks in combination with the semi-independence of levels of organization described above signifies that novel traits themselves are level-dependent. As new combinations of existing genes can elicit threshold effects, novel structures may appear without the introduction of novel genes. Similarly, the introduction of a novel gene does not guarantee a novel tissue or morphological structure. This does not suggest that there are never cases in which novelties are connected across multiple levels. Rather, it asserts that the introduction of novel traits does not require novelty at all levels.

An example of the discontinuous addition of a homonomous structure is the extra digits found in cats, particularly in the Maine Coon breed. The Hemingway Model (named for a genetic mutation underlying many cases of polydactyly in the famous cats in Ernest Hemingway’s former home) describes how a continuous distribution of cell states has thresholds leading to various discontinuous polydactyly states (Lange et al. 2014). In this model, a set of cells is influenced by the culmination of additive factors that determine the bistable state of individual cells (“on” or “off”). These states combine into a continuous variable distribution that can be mapped onto a set of thresholds determining the type of polydactyly present. This way a single point mutation, in a ~800 bp non-coding element that belongs to a cis-regulatory region driving the expression of sonic hedgehog, can result in the addition of 1–8 supernumerary digits in a single individual of Maine Coons (Lange et al. 2014).

These types of threshold traits indicate how novelties represent a link connecting quantitative/continuous variation with qualitative/discontinuous changes in the adult phenotype. One possibility of how this can occur is via biomechanical properties that impact developmental processes through mechanotransduction pathways. Mechanical forces are known to play a significant role in gene expression and development (Heegaard et al. 1999; Elder et al. 2000; van der Meulen and Huiskes 2002; West-Eberhard 2003; Keller et al. 2003; Vogel and Sheetz 2006; Haudenschild et al. 2009; Wozniak and Chen 2009; Grad et al. 2011), and these forces can cause tissues to undergo a state change if a threshold level is reached (Hall 1983; Müller and Streicher 1989; Vogel and Koob 1989; Tägil and Aspenberg 1999). For example, cyclic compressive force can elicit the differentiation of cartilage cells from the mesenchyme, whereas tension in connective tissue can initiate bone formation (Merrilees and Flint 1980; Carter and Wong 1988; Vogel and Koob 1989; Tägil and Aspenberg 1999; van der Meulen and Huiskes 2002; Hall 2005b; Nowlan et al. 2008; Kelly and Jacobs 2010; Grad et al. 2011).

A good example for the importance of mechanotransduction mechanisms can be found in the pharyngeal jaw apparatus of Cichlidae and Labridae (Peterson and Müller, submitted). These fish families have gained a novel synovial joint between the upper pharyngeal jaws (an extra set of jaws deep in the oral cavity) and the ventral surface of the neurocranium (Liem 1973). The introduction of this joint has occurred independently in both groups (Streelman and Karl 1997; Mabuchi et al. 2007), along with a set of derived traits in their pharyngeal jaw apparatuses that are functionally similar. These include a fused lower pharyngeal jaw, a new muscle sling connecting the lower pharyngeal jaw to the neurocranium, and a decoupling of the epibranchials 4 and pharyngobranchials (Liem 1973; Stiassny and Jensen 1987; Galis and Drucker 1996).

Some fish species have a single derived trait from this set without the others. In the cases of the fused lower jaw and the new muscle sling, the novel joint is not always present. However new joint is present when there is a decoupling of the epibranchials 4 and pharyngobranchials (Stiassny and Jensen 1987). Finite element analysis has shown that the fusion and new muscle sling result in no or minimal increase in force on the neurocranium from the upper pharyngeal jaws. In contrast, the decoupling can produce over four times as much force on the skull (Peterson and Müller, submitted). When the developing cichlid and labrid fry contract the muscles of the pharyngeal jaws, the pressure on connective tissue cells locally activates the genes required for cartilage formation, leading to a new joint at the point of contact between the neurocranium and the upper pharyngeal jaws.

This mechanism of developmental novelty origination makes use of gene networks that are already in place. Instead of relying on mutations directly creating a new tissue type or specifying the location for a novel structure, the new joint arises as a side effect of changes to other elements of the developmental system. This is possible due to the bottom-up nature of development, which relies on subroutines activated by local cues. Thus, evolutionary changes in the shape and configuration of tissues can alter the levels of stress and strain present in the system and cause genes to be expressed in locations where they were previously silent. These structural changes can be initiated by alterations in growth rate that change the orientations and magnitudes of muscle activity or relative tissue configurations, but the resultant change in biomechanical forces or tissue configurations, and therefore gene expression, cannot be deduced without charting the development and shape of the tissues involved. Furthermore, while the novel structure is a discontinuous change, it is usually a continuous input that triggers the crossing of the threshold, in this case force between two tissues.

Many examples exist of structures that originated through constructive development or discontinuous thresholds. T1 novelties can only be inferred from extant cell behaviors, but the T2 introductions of new tissue types and individualized elements have clear examples, and there are many well known cases of T3. Table 1 provides an overview of novelties that have been studied previously, along with justification for their categorization. The goal of this table is not to exhaust all known novelties or even those that have been studied more closely. Instead, it is used to show that a variety of processes can lead to discontinuous change, that there is diverse research on novelties of all types, and a wide range of both contemporary and historical research in this field is available.

External perturbations can also impact the developing organisms in discontinuous ways, and “it matters little from a developmental point of view whether the recurrent change we call a phenotypic novelty is induced by a mutation or by a factor in the environment” (West-Eberhard 2003). If the alteration persists due to the environment, it can become entrenched in development (Katz 1987) and eventually may become genetically assimilated (Waddington 1953). This relates to the idea of “genes as followers,” where a phenotypic structure that is present slowly accumulates genes that take on the role of refinement or ensure the production of a trait (West-Eberhard 2003; Schwander and Leimar 2011).

### Biomechanics in EvoDevo

**Fig. 1:**
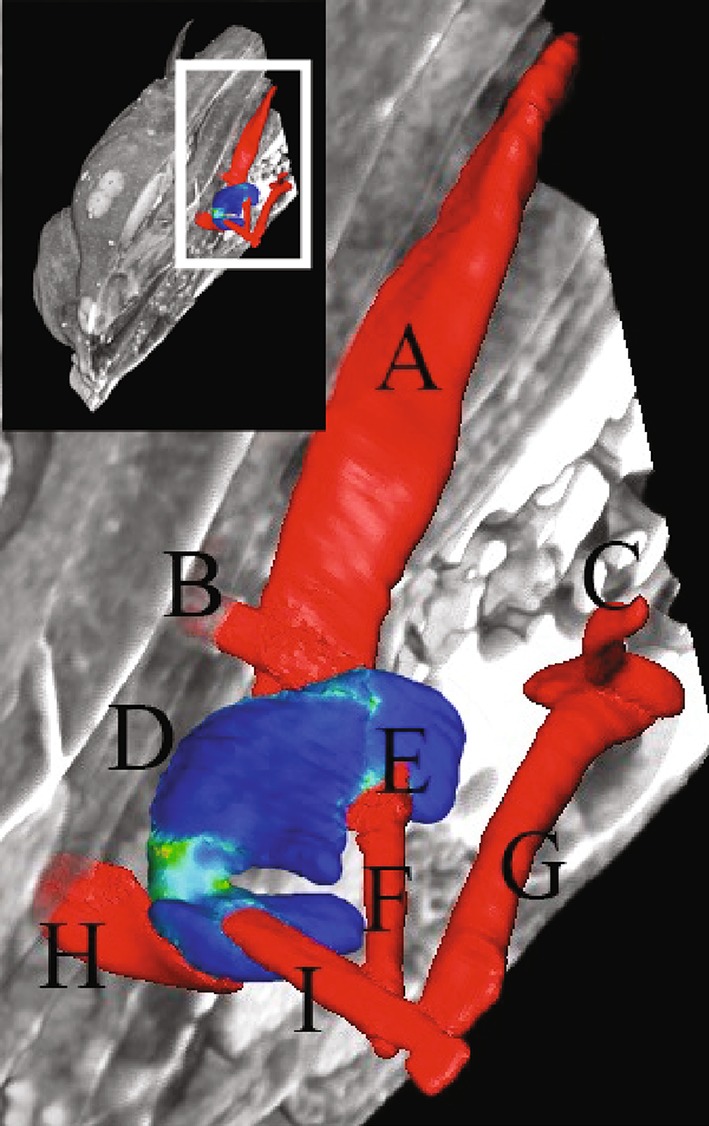
Finite element modeling of the upper pharyngeal jaw novelty in the cichlid *Amatitlania nigrofasciata.* A volume rendering of the fish, 6 days post fertilization, and associated musculature has been added to give spatial reference to the upper pharyngeal jaw. *Small window* The fish is shown slightly forward of the pectoral girdle, with one half cut away. Magnified area is outlined in *white*. *Large window* Muscles affecting pharyngeal jaw adduction are shown in red. The upper pharyngeal jaw is shown in *blues* and *greens* indicating various levels of von Mises stress. These types of models can determine the location, orientation, and magnitude of biomechanical signals during development. *a* retractor dorsalis; *b* transversus dorsalis posterior; *c* levator posterior; *d* upper pharyngeal jaw; *e* obliquus dorsalis; *f* levator internus lateralis; *g* levator externus 4; *h* transversus dorsalis anterior; *i* levator internus medialis

How can the described ways of developmental novelty generation be addressed by research in EvoDevo? One area previously mentioned and singled out here as an example of the physico-chemical aspect of development is biomechanics. To understand how the forces present during ontogeny can affect novelty formation, accurate 3D representations of various developmental stages in which the proposed mechanisms play out are needed. These can be obtained through techniques such as microCT scans or microMRI’s. Finite element analysis has recently begun to take off as a way to model physical forces in complex biological systems (Dumont et al. 2005; Ferrara et al. 2011; Goswami et al. 2011; Oldfield et al. 2012), and has the benefit of being compatible with a number of imaging software packages used with CT and MRI images, for example Amira (http://www.fei.com/software/amira-3d-for-life-sciences/) and 3Matic (http://software.materialise.com/3-maticSTL) that can create accurate models directly from scans of specimens. For analyses that require less precise representation these models can be manually created (Carter and Wong 1988). Finite element models enable the determination of force vector orientations and magnitudes, the stresses that occur because of these forces, the deformations on the structures, and the functional capabilities of the system (Fig. 1).

Another benefit of the modeling approach is that each model can be altered to represent hypothetical configurations (Peterson and Müller, submitted). This can be used to deduce the relative contribution of a derived trait, the interactions of multiple structures, where threshold events may have occurred, or the consequences of potential morphological changes. While not possible in all cases, various kinds of experiments can attempt to impede the development of a novelty, or induce it in closely related species, to verify model outcomes. This relies on experimental procedures, as well as comprehensive databases of embryonic material properties.

To complement these studies and 3D biomechanical–developmental atlases, a better understanding of mechanotransduction pathways will be required. This will involve determining the three-dimensional structures of each component involved using X-ray crystallography, NMP spectroscopy, and electron microscopy; and how the structural conformations change when force is applied to them. A set of articles highlights how the stress/strain curves for molecules can be obtained, with the protein Titin as an example, using three separate techniques: optical tweezers (Tskhovrebova et al. 1997), laser tweezers (Kellermayer et al. 1997), and atomic force microscopy (Rief et al. 1997). There are many other tests that can be done for determining if a tissue is even responsive to mechanical stimuli, though one of the most common is also the first one that demonstrated this: static stretch of a substrate in vitro (Vandenburgh and Kaufman 1979). Charting the mechanotransduction pathways and the force needed for their activation will aid in determining the time lag between stimulus and response that is needed to build proper models. These can be combined with studies at higher levels of organization that determine the force required on a tissue for a gene to be activated (Sadoshima et al. 1992; Takahashi et al. 1998; Tägil and Aspenberg 1999; Elder et al. 2000).

## Discontinuity in Phenotypic Change

Although organismal evolution generates discrete character states and does not occur at constant rates (Gould and Eldredge 1977; Krug and Jablonski 2012), it is still commonly considered to happen in small, incremental steps of quantitative variation of existing traits (Futuyma 2013). This is central to the adaptationist view, as it explains the gradual refinement of the phenotype by natural selection. Phenotypic deviations of large magnitude on a trait under selection have a greater chance of diverging from the optimum (Fisher 1930; Waxman 2006). Therefore, a set of small phenotypic changes is more likely to reach an adaptive peak than a single large change. Similarly, successful reproduction may be diminished by large-scale changes in the genome in sexually reproducing organisms due to incompatibilities between gametes. If changes in the phenotype occur along a different dimension than present in the trait under selection, increased adaptive value is even less likely to occur. While variation in new directions may be recognized (sometimes to unrealistic degrees that ignore developmental constraints), saltational changes of phenotypes have typically been rejected. Instead, large changes are seen as coming only from the extrapolation of variation over time (Lande 1980), with any gaps interpreted as an incompleteness of the fossil record (Gould and Lewontin 1979).

However, T2 novelties demonstrate that discontinuous changes in the adult phenotype can occur as a consequence of developmental dynamics rather than the accumulation of small variational changes. Since these qualitative changes are side effects of quantitative variation crossing a threshold, underlying genetic mutations do not need to be of a different type from those affecting quantitative variation, nor would any drastic change in chromosomal arrangements be required. Thus the discontinuous mode of change does not imply a genetic barrier to further breeding. While the trait may move the organism further away from an adaptive optimum, some subset of these novelties will be either adaptive or close enough to neutral to become fixed. Once established in a population, the novelty can be refined toward a new adaptive optimum (Erwin 2015).

The traits described herein are not akin to the often-maligned “hopeful monsters” of evolutionary history—hypothetical organisms with radically redesigned bodies or genomes—nor do they corroborate anti-Darwinian or teleological claims. Instead, they represent observable phenomena of phenotypic evolution, i.e. additions or alterations of tissues and structural traits: an added bone or joint, an invagination switching from the inside to the outside of the oral cavity, a new dermal shell or cuticle, etc. These are discontinuous traits in that they refer to a state change of a phenotypic trait between generations instead of the alteration of a quantifiable property along an axis of variation that was already present in the ancestral generation. If the discontinuous change is the addition or alteration of a phenotypic structure (instead of its loss), then it can be referred to as a novelty.

The recruitment of existing genetic networks into new locations in response to local cues, or the amplification of minor alterations in tissue configuration, allow for novel structures to form without large changes in the genome or the establishment of entirely new gene regulatory networks. Since these alterations take place at a higher organizing level than gene mutations, it is often unimportant which genes are causing the quantitative tissue change progressing towards the developmental threshold. Instead, the key point is that continuous variation of development is leading to a threshold response that results in a discontinuous phenotype. These neutral networks allow for a range of possible genetic mutations or interactions to exist that may produce a novel trait, increasing the likelihood of discontinuous change.

There are several possible pathways that allow for this type of discontinuous change to result from continuous variation. One possibility already mentioned involves biomechanical forces that initiate or inhibit the activation of mechanotransduction pathways. The formation of cartilage and bone are classic examples of development’s dependence on biomechanical forces (Hall 1986) and are also found in other tissues in both plants and animals (Sadoshima et al. 1992; Taber and Perucchio 2000; Keller et al. 2003; Chen and Brodland 2008; Adamo et al. 2009; Kuchen et al. 2012). Physical stresses rely on quantitative changes in structure shape, configuration of structures, muscle size, and muscle orientation. However, when these continuous alterations induce a mechanotransductive pathway to activate in a new location, the response is often a discontinuous change.

Another source of qualitative change from quantitative variation comes from morphogen gradients that work through “zero order ultrasensitivity” (Goldbeter 2005), negative feedback loops (Goldbeter et al. 2007), or reaction–diffusion systems (Turing 1952). One case that demonstrates the ability of morphogen gradients to produce discontinuous effects is the feedback between MAPK activity and Yan activation in the ventral ectoderm of *Drosophila* embryos that exhibits threshold characteristics (Melen et al. 2005). Certain pathway structures are more likely to lead to discontinuous changes than others and can be used as indicators of potential threshold effects. These pathways may be conserved despite co-option or alterations of their components. A good example of pathway structure retention despite component divergence is the receptor tyrosine kinase-ras signal transduction pathway. The pathway is so well conserved that its components are interchangeable between mice, nematodes, and fruit flies (Downward 1994; Gilbert et al. 1996). All of these examples show discontinuous events in development that can be co-opted to create novel morphological structures.

Several modes of discontinuity generation do not rely on developmental threshold events. For instance, the co-option of an existing gene regulatory network, including the alteration of a *cis*-regulatory element resulting in gene expression at a new location, also has the potential to promptly insert a novel tissue or phenotypic structure into a new location (Rebeiz et al. 2011). In these cases, while there is still a discontinuous change, the novelty is not the result of a threshold effect. However, large phenotypic changes that result from such an event may be reliant on the plasticity of the developmental system to remain integrated and viable. New tissue types, such as the introduction of photoreceptive cells, may also have discontinuous origins that are not related to threshold events.

Small quantitative changes, regardless of their origin, may still result in a discontinuous structure if they are amplified due to developmental cascades, particularly if they give cues for future developmental processes. One example of this is the external cheek pouches in some species of rodent. The pouches begin with a small invagination that progresses into a pouch. In most species, this expansion occurs within the buccal cavity. However, a selectional change in facial proportion adjusts the starting location of an invagination and induces external cheek pouches to form (Brylski and Hall 1988a, b). Hence, altering continuous underlying developmental variation to a threshold point will result in a discontinuous trait. In contrast to the internal pouches, these external pouches will also be lined with fur, as local cues control much of development and fur induction is a pre-existing mode in the immediate vicinity of the new pouch.

Similarly, downstream events cause a tubular feather follicle to form instead of a planar scale due to the initial condition of an increase of cells in a ring around an epidermal placode (Prum and Brush 2002). While both structures begin in development as an epidermal thickening, continuous variation among adults of subsequent generations would be exhibited by the thinning of the sides of scales, which might then be followed by their branching to create the barbules of the feather, while the inner portion stayed thick and became the rachis. However, “from their origin within the follicle until final emergence, all feathers are cylindrical” and “the dorsal and ventral surface of a mature feather are created by the peripheral and inner surfaces of the follicle collar… cannot be considered homologous with the dorsal and ventral surfaces of a scale” (Prum 1999). Instead, the continuous changes to the underlying primordium lead to a discontinuous change in the adult definitive structure: planar to tubular.

Furthermore, while mutations may occur randomly, the resultant structure is determined by non-random developmental rules. This inserts a degree of predictability for the appearance of some novel characters that enables the testing of the developmental rules through perturbation events in ontogeny. Thus it is possible for EvoDevo to identify areas of potential future novelties, particularly in combination with ecological and population genetic tools. While it is well accepted that natural selection acting on small quantitative changes is the predominant mode for the refinement of existing structures, a number of traits have developed discontinuously. These “large steps” are increasingly considered an important feature in evolution (Frazzetta 2011). These novel traits (see Table 1 for an incomplete list) are considered discontinuous because the resultant phenotypic structure does not have a homologous counterpart in the ancestor. While initiated through quantitative changes in development, the end product often is a binary switch between absent and present, instead of the quantitative alteration of variables that already exist for an established trait.

In this way, threshold events in development connect discontinuous novel traits to continuous variation in the existing underlying parameters. A developmental system can be quantitatively altered to the point that epigenetic cues initiate new developmental trajectories leading to qualitatively different structures. These traits do not therefore contradict the importance of heritable variation and natural selection. Instead, they show that the bottom-up processes of development can also generate discontinuous novelties.

**Fig. 2:**
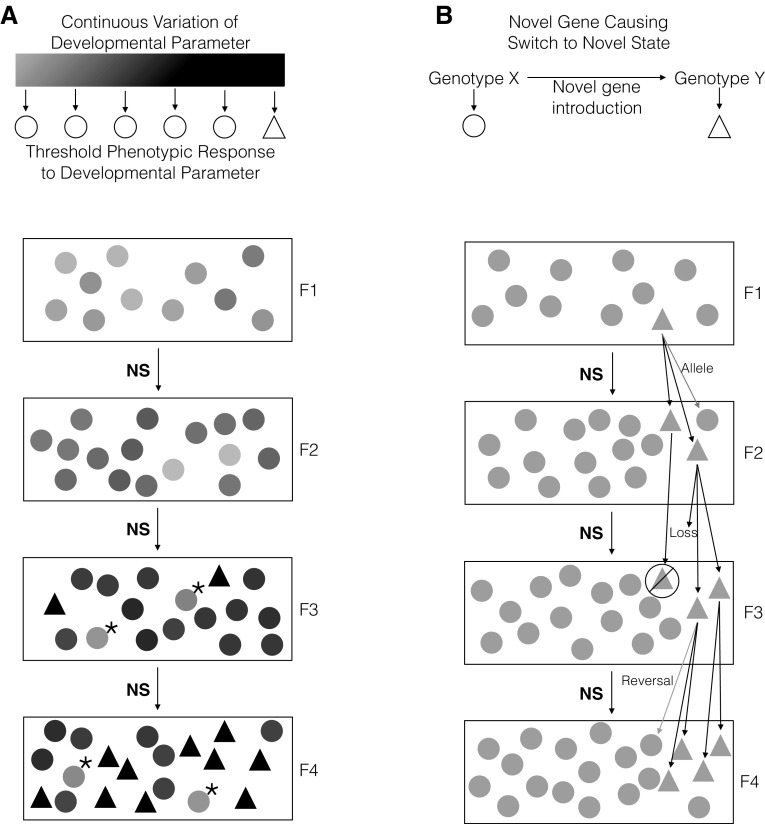
Population level spread of a discontinuous trait (**a**) versus spread from a single (mutant) individual (**b**). *Circles* represent individuals without the novelty; *triangles* represent individuals with the novelty. The figure demonstrates hypothetical general patterns of novelty origination and is not meant to represent accurate ratios of novelty versus ancestral traits, likelihood of events, etc. **a** A developmental parameter can undergo continuous variation, represented here as variable grey scale value, which leads to the same adult structure, represented here as a *circle*. If the variation crosses a threshold level, a discontinuous change resulting in a qualitatively new phenotype occurs, represented here as a *triangle*. As the novelty is determined by the developmental system and is in another dimension than the threshold that creates it, their symbols are purposely incommensurable (*shapes and colors*). In each successive generation, natural selection pushes the population towards the threshold (such as a biomechanical signal, morphogen patterning process, etc.) shown by the *circles* becoming darker. By F3, some individuals have crossed the threshold. By F4, more individuals have crossed the threshold. Since the threshold is determined by properties of the phenotype and does not necessarily depend on one particular gene, many different gene combinations may be involved in passing the threshold. Loss of individual genetic lines through death, no mating, etc., do not hinder the spread of the novelty as other individuals are close to the same threshold. Similarly, variation away from the threshold (represented by *asterisk*) does not put the entire population at risk for loss of the novelty. Importantly, the critical threshold is approached only by coincidence, and the selection pressure on the trait is unrelated to the existence of a threshold. **b** Origination of novelty from a specific gene mutation is spread only from the individual with the mutation, and relies on positive selection for that novelty as opposed to selection on another trait that has a threshold. Several factors can cause loss of the trait. “Allele”: Offspring may inherit the allele without the novel mutation. “Loss”: Individuals with the novel mutation may die during development or before they have a chance to mate. “No symbol”: Inability to produce viable offspring or find a mate. “Reversal”: Individuals may inherit the novel mutation, but in a different genetic background it may not result in the novel phenotype. The latter possibility may allow the gene to be retained in the gene pool, but there is no selection pressure for its maintenance. The other possibilities remove the gene. In the initial generations after the gene mutation, the small number of individuals with the mutation and the large number of ways in which it can be lost make it less likely to be spread than in the population level dynamic shown in **a**. Critically, case **b** requires positive selection (or drift) on a novel gene that is able to spread throughout a population, while case **a** has the entire population primed for the introduction of a novelty, which can occur with the genes already present in the gene pool

Since natural selection can cause a whole population to approach a relevant threshold, variation within the population may cause multiple individuals to simultaneously express the T2 novelty (or lose it) under similar selective or environmental conditions (West-Eberhard 2003; Jaeger et al. 2012). The new phenotype can then be selected for, resulting in a reinforcement of the local cues that create the novelty, or against, reducing variation that may cause the threshold to be crossed. However, the threshold is initially approached by coincidence, and the selection pressure on the trait is unrelated to the existence of a threshold. In the case of positive selection, T2 novelties caused by threshold effects may spread more easily than novel genes, which require inheritance from a single individual and risk loss due to beneficial allele combinations being broken up during sexual reproduction or through chance death of the initial individual carrying the mutation. With the population as a whole being driven by natural selection towards a threshold, there is no risk associated with the loss of a single individual or loss of the trait due to a different genetic background as there is with explaining novelties through gene mutations, since multiple individuals will have the same potential to develop the novelty (Fig. 2).

## Novelties, EvoDevo, and an Extended Evolutionary Synthesis

The phenotypic novelty concept shows how processes that take place during ontogeny have generative potential for structures that become important for the evolution of the species. Large-scale changes do not come solely from the accumulation of small-scale, continuous variation. Instead, the bottom-up procedures of the developmental system can generate novelty at the phenotypic level. While these innovation events may be more rare than the typical shifting of allele frequencies or the introduction of a new allele or gene through mutation, they can have a profound influence on evolutionary trajectories. This is particularly true if the novel structure becomes a key innovation that enables new (possibly) adaptive radiation and can be modified in a number of ways or makes a functional group more modular to allow for diversification (Liem 1973). While the direction of phenotypic evolution is typically accounted for by natural selection on gene pools in standard scenarios, the influence of developmental systems on biasing phenotypic evolution, inserting new elements, and determining their form lend explanatory power to EvoDevo as well (Laland et al. 2015).

The number of mechanistically different ways of innovation creates a wide range of approaches to investigate novelty generation. This article focused on biomechanics, including advocacy for the creation of biomechanical developmental atlases paired with research on mechanotransduction pathways. Other avenues include research on generic patterning processes, cell signaling, and the developmental cascades in tissue organization. In addition, the dynamics of gene regulatory network evolution in natural populations hold important implications for understanding how they facilitate the generation of phenotypic novelty and how development mediates the response of organisms to environmental change (Favé et al. 2015). Computer simulations of gene regulatory networks show how discontinuous changes in the phenotype may be more likely to occur than quantitative variational change when the network changes its dynamical behavior, with quantitative change only exhibited when there is a shift in the attractor point of a network (Jaeger et al. 2012). Together, these various approaches share a focus on the mechanisms of morphological evolution and novelty generation not seen in other fields. The conceptual consequences of this kind of EvoDevo research contribute to alternative theoretical frameworks of evolution, such as the Extended Evolutionary Synthesis (Pigliucci and Müller 2010; Laland et al. 2015).

**Fig. 3:**
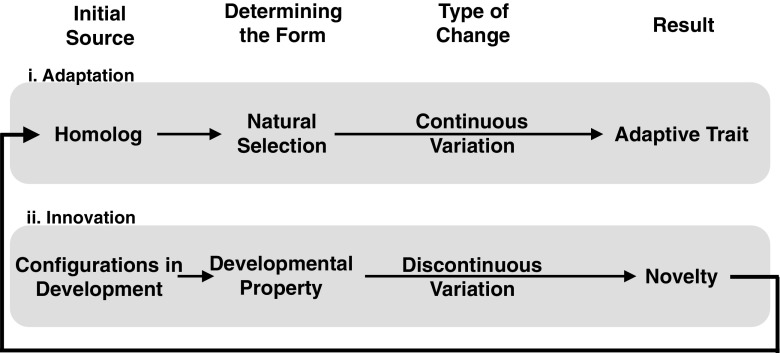
Schematic connecting the processes of innovation and adaptation. **i** Adaptation. A preexisting element is the starting source for adaptive change. Natural selection acting on heritable variation determines the form of the phenotype. This works through continuous variation, with small changes in each generation resulting in an adaptive trait present in the population. **ii** Innovation. The initial source for innovation is the configuration of the developmental system, including both epigenetic and genetic factors. Epigenetic in this case refers to traits and processes above the gene level, such as environmental factors, tissue interactions, biomechanical forces, etc. A developmental property, such as cartilage induction by compression, determines the form that occurs from the developmental configuration. In the case of novelties, this form appears as discontinuous variation of the phenotype compared to previous generations. The resulting novelty, a new homologue, can undergo further adaptation. Part **i** represents the *striped borders* and part **ii** the *solid black borders* in Fig. 4

Another theoretical consequence is the change of explanatory roles for natural selection and development in phenotypic evolution. In the received view, natural selection refines the genetic underpinning of phenotypic structures and body plans, and development is merely the expression of the genetic outcome of selection. This has led to criticism that development is being black boxed in the standard theoretical accounts (Weydert 2004; Hendrikse et al. 2007). However, in the case of novelties, natural selection can drive a species toward a developmental threshold, but the resultant phenotype is not a direct consequence of the refinement by natural selection. Instead, the developmental system determines what structures arise and how the body plan accommodates the introduction of a novelty. Only after a trait is present in a rudimentary form, and if its expression contains some variation that can be selected on, the population genetic mode of variation may take over to refine a novelty (Fig. 3). This highlights how seemingly similar events are shaped by different factors in innovation and adaptation. While the straightforward paradigm of natural selection acting on variation and resulting in a matching between phenotype and environment may be sufficient to explain variational change of established structures, the causality for the origination of new structures or elements in a body plan lies in the properties of the affected developmental system.

**Fig. 4:**
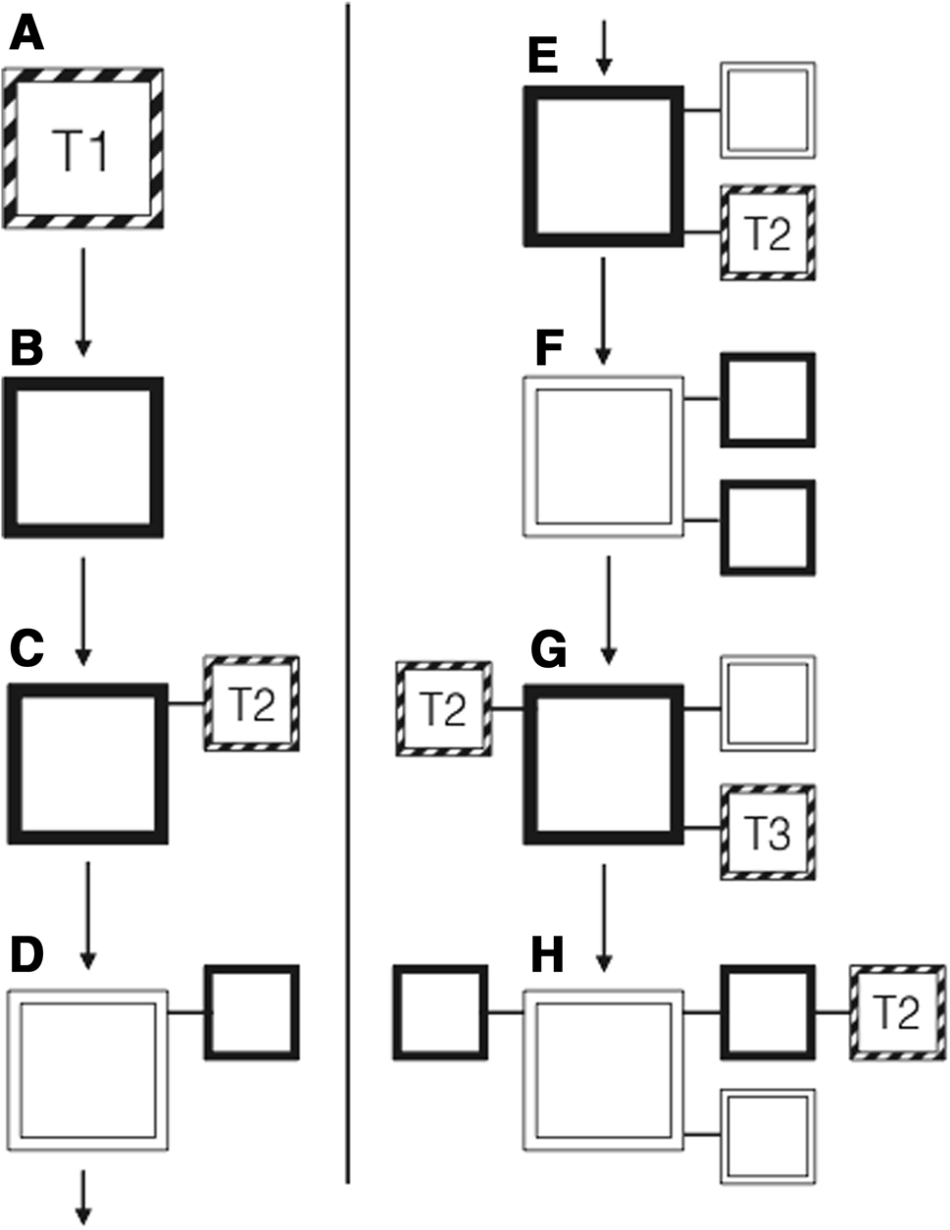
Schematic for the evolutionary introduction of three kinds of phenotypic novelties concurrent with variational change. Each section of A-H represents a single individual in a population that is experiencing morphological evolution, with each sequential section representing the next morphology. The type of evolution of a single structure is denoted by *striped*, *white*, or *black outlines*. *Striped outlines* indicate qualitatively discontinuous phenotypic change arising in a direction determined by the developmental system. This corresponds to Fig. 3 **ii** (Innovation). *Solid black outlines* indicate quantitative phenotypic change determined by natural selection. This corresponds to Fig. 3 **i** (Adaptation). White borders show phenotypic stasis. **a** Establishment of a new multicellular body assembly, a T1 novelty. **b** Body plan undergoes quantitative change. **c** Body plan continues to undergo quantitative change, while a qualitatively new structural unit, a T2 novelty, is added. **d** Body plan is stasis while the new structure undergoes quantitative change. **e** Body plan evolves quantitatively, structural unit is in stasis, and a new T2 unit is added to the body plan. **f** Body plan is in stasis, both structural units are undergoing quantitative change. **g** Body plan evolves quantitatively, a new structural unit is added, the first structural unit is in stasis, and the second structural unit has quantitative change in a qualitatively new dimension, a T3 novelty. **h** Body plan is in stasis. The first structural unit undergoes quantitative change, and passes a threshold to create a T2 novelty. The second structural unit is in stasis. The third structural unit experiences quantitative change

In the light of these findings, and despite perpetuated assertions to the contrary (Futuyma 2013), microevolutionary events are insufficient for explaining discontinuous forms of change and phenotypic novelties. The idea that small, continuous, incremental variational change is the sole cause of phenotypic evolution continues to be challenged by qualitatively discontinuous changes that also need to be accounted for by evolutionary theory (Müller and Wagner 1991; Salazar-Ciudad and Jernvall 2005; Pigliucci and Müller 2010; Frazzetta 2011; Jaeger et al. 2012; Peterson and Müller 2013; Linde-Medina and Newman 2014). This is different from debates on the rates of evolution and instead comments on the types of changes possible. Stephen Jay Gould contrasted ideas of how evolution proceeds by comparing it to either a billiard ball or what he termed Galton’s polyhedron (Gould 1988). In this view, evolution proceeds between flat areas of stasis, the faces of the polyhedron, interrupted by large shifts as it tumbles over the polyhedron’s edges. Gould famously argued “organisms are not billiard balls, struck in deterministic fashion by the cue of natural selection, and rolling to optimal positions on life’s table. They influence their own destiny in interesting, complex, and comprehensible ways.” Through EvoDevo it is now possible to determine, describe, and study these “comprehensible ways.” Gould had invoked the influence organisms assume over their own evolution, through processes such as developmental bias and constraint (Müller 2013). Even though phenotypic change is pushed forward by natural selection, forces outside of selection often control the direction of these changes. These “forces” can be more aptly described as the developmental system. It holds the potential for explaining discontinuous change, because evolution neither proceeds exclusively according to the adaptation–selection view of a billiard ball driven by natural selection to roll continuously to the next form, nor by Galton’s polyhedron switching discontinuously between stable states, but rather as a combination of both acting at the same time (Fig. 4). As Laland and colleagues have argued (Laland et al. 2015), a new and more comprehensive framework of evolutionary theory needs to include, among other components, a concrete and potentially formalizable account of EvoDevo mechanisms.
